# GAIT SLOWING AMONG FRAIL OLDER ADULTS: IS HIGHER DOPAMINERGIC SIGNALING PROTECTIVE?

**DOI:** 10.1093/geroni/igz038.340

**Published:** 2019-11-08

**Authors:** Caterina Rosano, Stephanie Studenski, Nicolaas Bohnen, Andrea Rosso

**Affiliations:** 1 University of Pittsburgh, PIttsburgh, Pennsylvania, United States; 2 NIA, Longitudinal Studies Section, Baltimore, Maryland, United States; 3 University of Michigan, Ann Arbor, Michigan, United States; 4 University of Pittsburgh, Department of Epidemiology, Pittsburgh, Pennsylvania, United States

## Abstract

Strategies to reduce gait slowing in frail older adults are urgently needed. Higher dopaminergic (DA) signaling is emerging as a protecting factor against age-related gait slowing, in the absence of Parkinson’s Disease (PD). DA signaling is potentially modifiable, thereby offering promising novel strategies to reduce gait slowing. In 3,752 PD-free participants of the Cardiovascular Health Study (72.3 years, 81% white, 39% male), we measured gait speed (usual pace, 15 feet), frailty (Fried definition), and genetic polymorphism of Catechol-O-methyltransferase (COMT, rs4680), an enzyme regulating tonic brain DA levels. Multivariable linear regression models of COMT predicting gait speed were adjusted for age, gender, BMI, ankle-arm index, vision, and arthritis. Strength, education, medications, pulmonary, cardio- and cerebro-vascular diseases, diabetes, mood, and cognition were considered as additional covariates. We examined the full cohort and the subgroup with frailty (n=222), without and with race-stratification to address racial differences in allele frequencies. Average (SE) gait speed was 0.88 (0.003) and 0.58 (0.01) m/sec in the full cohort and the frail subgroup, respectively. COMT was linearly associated with gait speed; gait was faster for met/met (higher DA signaling) and slower for val/val (lower DA signaling) participants. In adjusted models, differences between these two groups were: 0.02 (0.01) m/sec in the full cohort (p=0.4); 0.07(0.02) m/sec in the frail subgroup (p=0.02); 0.10 (0.02) m/sec in white with frailty (p=0.01). COMT genotyping may help identify frail adults who are less vulnerable to gait impairments. Studies of frailty should examine whether higher DA signaling offers resilience against age-related gait slowing.

